# WSF: A Transformer-Based Framework for Microphenotyping and Genetic Analyzing of Wheat Stomatal Traits

**DOI:** 10.3390/plants14193016

**Published:** 2025-09-29

**Authors:** Honghao Zhou, Haijiang Min, Shaowei Liang, Bingxi Qin, Qi Sun, Zijun Pei, Qiuxiao Pan, Xiao Wang, Jian Cai, Qin Zhou, Yingxin Zhong, Mei Huang, Dong Jiang, Jiawei Chen, Qing Li

**Affiliations:** 1 Sanya Institute, Nanjing Agricultural University, Sanya 572000, China; honghao_zhou@stu.njau.edu.cn (H.Z.); 2025801239@stu.njau.edu.cn (Z.P.); 2 Academy for Advanced Interdisciplinary Studies, Nanjing Agricultural University, Nanjing 210095, China; 2024222001@stu.njau.edu.cn; 3 National Technique Innovation Center for Regional Wheat Production/Key Laboratory of Crop Ecophysiology, Collaborative Innovation Centre for Modern Crop Production, Ministry of Agriculture and Rural Affairs/College of Agriculture, Nanjing Agricultural University, Nanjing 210095, China; 2023101025@stu.njau.edu.cn (H.M.); 2023801279@stu.njau.edu.cn (S.L.); 2025801323@stu.njau.edu.cn (Q.S.); xiaowang@njau.edu.cn (X.W.); caijian@njau.edu.cn (J.C.); qinzhou@njau.edu.cn (Q.Z.); yingxinzhong@njau.edu.cn (Y.Z.); huangmei@njau.edu.cn (M.H.); 4 Shandong Hedian Agricultural Technology Co., Ltd., Jinan 250000, China; 13287952369@163.com

**Keywords:** wheat, stomata, deep learning, semantic segmentation, stomatal traits, genome wide association study

## Abstract

Stomata on the leaves of wheat serve as important gateways for gas exchange with the external environment. Their morphological characteristics, such as size and density, are closely related to physiological processes like photosynthesis and transpiration. However, due to the limitations of existing analysis methods, the efficiency of analyzing and mining stomatal phenotypes and their associated genes still requires improvement. To enhance the accuracy and efficiency of stomatal phenotype traits analysis and to uncover the related key genes, this study selected 210 wheat varieties. A novel semantic segmentation model based on transformer for wheat stomata, called Wheat Stoma Former (WSF), was proposed. This model enables fully automated and highly efficient stomatal mask extraction and accurately analyzes phenotypic traits such as the length, width, area, and number of stomata on both the adaxial (Ad) and abaxial (Ab) surfaces of wheat leaves based on the mask images. The model evaluation results indicate that coefficients of determination (R^2^) between the predicted values and the actual measurements for stomatal length, width, area, and number were 0.88, 0.86, 0.81, and 0.93, respectively, demonstrating the model’s high precision and effectiveness in stomatal phenotypic trait analysis. The phenotypic data were combined with sequencing data from the wheat 660 K SNP chip and subjected to a genome-wide association study (GWAS) to analyze the genetic basis of stomatal traits, including length, width, and number, on both adaxial and abaxial surfaces. A total of 36 SNP peak loci significantly associated with stomatal traits were identified. Through candidate gene identification and functional analysis, two genes—*TraesCS2B02G178000* (on chromosome 2B, related to stomatal number on the abaxial surface) and *TraesCS6A02G290600* (on chromosome 6A, related to stomatal length on the adaxial surface)—were found to be associated with stomatal traits involved in regulating stomatal movement and closure, respectively. In conclusion, our WSF model demonstrates valuable advances in accurate and efficient stomatal phenotyping for locating genes related to stomatal traits in wheat and provides breeders with accurate phenotypic data for the selection and breeding of water-efficient wheat varieties.

## 1. Introduction

Wheat holds a crucial position in China’s grain production and is one of the country’s main staple crops. The development of the wheat industry is directly related to national food security and social stability. Therefore, improving wheat yield is of significant importance for ensuring food security and promoting farmers’ income in China [1]. Stomata are the main channels for the exchange of substances between wheat and the external environment. Their morphological traits, such as size and density, directly affect physiological activities such as photosynthesis and transpiration in the leaves, determining the wheat’s material yield and drought resistance [2,3]. Therefore, analyzing the genetic mechanisms of wheat stomatal traits is of great scientific value for studying wheat drought resistance mechanisms and for drought-resistant breeding.

Unlike most dicotyledonous plants, where stomata can be directly observed by peeling off epidermal cells, monocotyledonous plants such as wheat have a thinner mesophyll layer, making the peeling of epidermal cells very difficult [4]. Therefore, appropriate fixatives are often used to preserve the stomatal morphology, followed by microscopic observation, or stomata can be directly observed under a microscope [5]. However, the process of identifying and counting stomata through microscope images is cumbersome. After capturing the images, manual identification and counting of stomata are required, which is time-consuming and labor-intensive. Long-term reliance on visual observation also increases the likelihood of errors. With advancements in technology, image processing software such as ImageJ-154 [6] has been used to semi-automatically identify stomata and measure their length and width, but this process still requires users to manually mark the boundaries of stomata and other points of interest [7]. This method has certain limitations in terms of accuracy, speed, throughput, and automation, and cannot meet the modern scientific demand for efficient stomatal data acquisition. Therefore, developing a non-destructive method capable of quickly collecting plant stomatal data will significantly improve the efficiency of subsequent research and drive progress in related fields.

With the rapid development of deep learning, the automatic detection and recognition technology for plant stomata has greatly advanced [8], significantly improving the efficiency of stomatal research. In stomatal studies, deep learning methods can be applied for automatic detection, counting, and complex feature extraction, providing researchers with more comprehensive and accurate data. Currently, deep learning methods have been applied in stomatal trait detection. Stomata Counter, a stomatal counting tool based on the R-CNN deep convolutional neural network, has achieved an accuracy of 0.98 for ginkgo stomata recognition [9]; in maize crop live stomata image recognition, a stomatal counting model based on the YOLO framework [10] achieved a precision of 0.89 [11,12]. However, the models mentioned above are all object detection models, which use bounding boxes to mark the regions where stomata are located. However, they still rely on subsequent image processing steps to extract the stomatal pixels within the bounding boxes. This traditional approach has certain computational errors, and for small targets such as stomata, the bounding boxes often fail to capture their details precisely, making it difficult to reflect the actual morphology of small targets. Semantic segmentation models [13], which can finely classify each pixel in an image, are capable of accurately extracting stomatal regions. These models not only delineate the contour boundaries of stomata but also generate high-quality masks, providing precise support for monitoring stomatal morphological changes, especially for target tracking and phenotypic quantification analysis in dynamic processes. The Mask RCNN model [14] has shown ideal results in stomatal complex segmentation and classification in multiple tree species [15,16]; the UNet model [17] has been used to extract contour coordinates of pore regions from wheat leaf microscopic images and to calculate the morphological characteristics of stomata and pores based on these coordinates [18]. However, both Mask-RCNN and UNet have issues in wheat stomatal segmentation, including high computational complexity, insufficient boundary accuracy, and weak long-range dependency modeling capability, especially when dealing with small targets. In contrast, SegFormer [19], through the self-attention mechanism, captures global contextual information, improves boundary precision for small targets, and provides higher computational efficiency and robustness, making it particularly suitable for fine segmentation of stomata in complex backgrounds, which is ideal for the stomatal segmentation task in this study.

Genome-wide association studies (GWAS) identify genetic variations closely related to specific traits by comparing genomic variations with phenotypic characteristics in different individuals [20]. Currently, GWAS has been widely applied in the development of linkage markers and gene mining for crops such as rice [21], maize [22], wheat [23], and barley [24]. However, in these GWAS studies, phenotypic traits are usually measured manually, which is time-consuming and prone to subjective errors. With the application of deep learning methods, stomatal boundaries or masks can be efficiently extracted, and accurate morphological traits related to stomata can be obtained. These traits reflect the plant’s growth status and adaptability under different environmental conditions [25]. Combining deep learning-extracted phenotypic data with GWAS not only improves the efficiency and accuracy of trait acquisition but also helps uncover the genetic mechanisms of complex agronomic traits. Through this deep learning-assisted research, researchers can study traditional traits such as disease resistance and yield, while simultaneously analyzing multiple traits of crops, including those that cannot be obtained through manual examination, leading to the identification of numerous significantly associated loci and the discovery of more related genes.

To address the current challenges in stomatal research related to efficiency and automation, this study aims to propose an innovative solution, focusing on non-destructive methods for acquiring stomatal images from wheat leaves of multiple varieties and developing a stomatal semantic segmentation method, Wheat Stoma Former (WSF), that is applicable to different species. This method integrates deep learning techniques to extract key phenotypic parameters and conducts genome-wide association studies based on phenotypic data. Specifically, the research objectives include the following three aspects: first, to construct a high-quality non-destructive wheat leaf image dataset, using high-precision imaging devices to capture stomatal samples from multiple varieties, providing a foundation for subsequent modeling; second, to design and implement a universal stomatal segmentation framework, WSF, applicable to multiple species, where the integrated Cross-Layer Feature Pyramid Module (CFPT) effectively suppresses background noise interference while enhancing edge structure extraction, thereby improving segmentation accuracy; finally, to conduct GWAS on 210 wheat varieties using the extracted stomatal phenotypic parameters, identify significant SNP loci closely related to stomatal traits, and recognize potential regulatory genes. This will provide theoretical support and data for understanding the genetic basis of stomatal traits and their applications in breeding. This research framework ensures both data acquisition efficiency and accuracy while balancing general applicability and practicality, offering the potential to advance the integration of crop phenotypic intelligent analysis and molecular breeding research.

## 2. Results

### 2.1. Model Performance Testing and Comparative Experiments

To verify the effectiveness of the proposed WSF model, we selected two mainstream semantic segmentation architectures, Deeplabv3+ and UNet, as baseline models for comparative experiments. All models were trained under consistent training configurations to ensure fairness, and segmentation performance was quantitatively assessed using the Precision and Mean Intersection over Union (MIoU) metrics (Table 1). The experimental results clearly show that the WSF model exhibits significant advantages in the stomatal mask segmentation task across multiple crops. Its performance in both accuracy and MIoU exceeds that of the comparison models, demonstrating stronger feature extraction capability and generalization performance.

**Figure 1:**
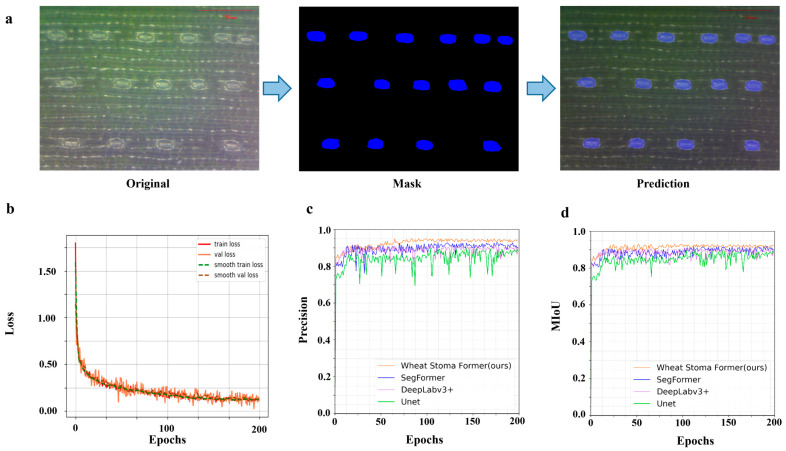
Model segmentation performance testing and parameter variation of the WSF model during the training process. (The blue area is the stomatal mask segmented by the model.) (**a**) The segmentation results of wheat stomata. (**b**) Training and testing loss curve image. (**c**) Image of precision variations of different models. (**d**) Image of MIoU variations of different models.

This section presents systematic experiments to validate the practical application of the WSF model in the stomata segmentation of wheat (Figure 1a), highlighting the effectiveness of the proposed model in the application of wheat stomatal semantic segmentation. To further improve training efficiency and accelerate convergence, we adopted a dynamic learning rate optimization strategy. This strategy automatically adjusts the learning rate based on the progress of the current training. For example, when the loss decreases at a slower rate during a certain stage of training, the learning rate is moderately reduced, which prevents the model from getting stuck in local minima and helps maintain convergence stability. This dynamic adjustment of the learning rate improves the model’s performance in the later stages of training and prevents volatility caused by excessively large learning rates.

To better understand the model’s performance during training, we saved four key metrics at each training batch: training loss, validation loss, smoothed training loss, and smoothed validation loss. Training loss refers to the error of the model on the training set, reflecting how well the model fits the training data. Validation loss refers to the error of the model on the validation set, reflecting the model’s generalization ability, i.e., how well the model can adapt to unseen data. Smoothed training loss and smoothed validation loss are versions of the original loss curves that have been smoothed to observe the trend of the model more clearly and avoid interference from fluctuations between batches in assessing the model’s learning progress. The initial training epoch was set to 200, and by using the backpropagation (BP) algorithm to optimize the loss function, we ensured that the model could effectively learn the spatial features and morphological information of wheat stomata. To accelerate convergence and improve training efficiency, we combined the dynamic learning rate optimization strategy, which automatically adjusts the learning rate based on the loss values during the training process, ensuring more stable convergence in the later stages of training. During the training, we saved the training loss and validation loss for each batch in real time and visualized both the original and smoothed losses (Figure 1b) to intuitively monitor the model’s training progress and performance changes. With these optimization strategies, the model’s final training loss stabilized around 0.15, indicating that the model has adequately fit the training data and demonstrated strong generalization ability on the validation set.

The changes in precision and MIoU of the WSF model at each training epoch are shown below (Figure 1c,d). From the graphs, it can be clearly observed that the model not only shows faster convergence but also possesses significantly higher training stability. Specifically, the WSF model reaches a performance plateau after approximately 50 epochs, with the precision and mIoU curves stabilizing thereafter. In contrast, the SegFormer model requires around 80 epochs to reach relative stability, while Deeplabv3+ requires up to 150 epochs to converge. More notably, the traditional UNet model exhibits large fluctuations throughout the entire training process, showing clear instability.

This training advantage is attributed to the optimizations in the WSF model. First, in terms of model structure, it integrates a multi-scale feature extraction mechanism and a lightweight attention module, enabling it to accurately capture edge and texture features of stomatal regions at different scales, especially for complex multi-crop stomatal morphological backgrounds. Second, in terms of training optimization, we adopted dynamic learning rate adjustment and a hybrid loss function design, effectively reducing the risk of overfitting and accelerating the convergence of model parameters. Additionally, the introduction of data augmentation and noise robustness mechanisms further enhanced the model’s generalization ability under different lighting, resolution, and tissue structure backgrounds.

Overall, the WSF model outperforms existing mainstream models in terms of precision and MIoU and also exhibits excellent performance in training efficiency and stability. This performance demonstrates its broad applicability and potential for engineering deployment in high-throughput plant phenotyping scenarios, particularly in crop phenotyping research that requires high precision for stomatal localization and recognition.

### 2.2. Stomatal Trait Accuracy Assessment

**Figure 2:**
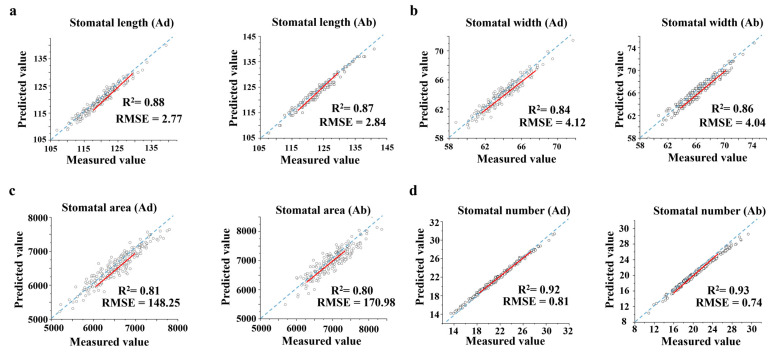
Stomatal trait regression comparison graph. Each genotype had 3–4 replicates. (The red lines are the fitted line segments.) (**a**) Fitting result of stomatal length. (**b**) Fitting result of stomatal width. (**c**) Fitting result of stomatal area. (**d**) Fitting result of stomatal number.

This study validated and compared the stomata of 210 wheat varieties. The accuracy of the algorithm was evaluated using two metrics: coefficient of determination (R^2^) and root mean square error (RMSE). The manual measurement values were obtained using the ImageJ-154 tool software for assistance, and the comparison between the manual measurement values and system-estimated values of the phenotypic parameters is shown in Figure 2. For stomatal length (Figure 2a), the R^2^ for the system-estimated stomatal length on the adaxial and abaxial surfaces with manual measurements were 0.88 and 0.87, with RMSE of 2.77 pixels and 2.84 pixels, respectively. For stomatal width (Figure 2b), the R^2^ for the system-estimated stomatal width on the adaxial and abaxial surfaces with manual measurements were 0.84 and 0.86, with RMSE of 4.12 pixels and 4.04 pixels, respectively. For stomatal area (Figure 2c), the R^2^ for the system-estimated stomatal area on the adaxial and abaxial surfaces with manual measurements were 0.81 and 0.80, with RMSE of 148.25 pixels^2^ and 170.98 pixels^2^, respectively. For stomatal number (Figure 2d), the R^2^ for the system-estimated stomatal number on the adaxial and abaxial surfaces with manual measurements were 0.92 and 0.93, with RMSE of 0.81 and 0.74, respectively. The correlation between the system’s predictions and the traditional ImageJ manual measurements is highly significant, especially for stomatal number prediction, demonstrating the value of this method in agricultural phenomics research.

### 2.3. Analysis of Stomatal Differences Between the Adaxial and Abaxial Surfaces

**Figure 3:**
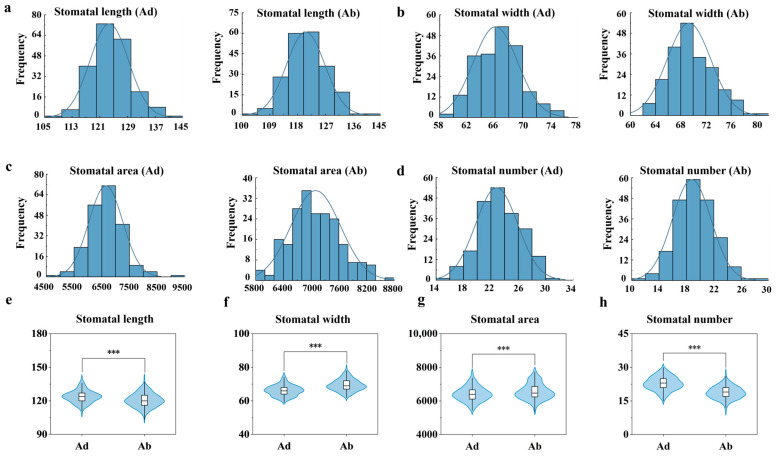
Frequency distributions and violin plots of four stomatal traits, based on data from three replicates of 210 wheat varieties. (**a**) Frequency distribution diagram of stomatal length. (**b**) Frequency distribution diagram of stomatal width. (**c**) Frequency distribution diagram of stomatal area. (**d**) Frequency distribution diagram of stomatal number. (**e**) Violin plot of stomatal length. (**f**) Violin plot of stomatal width. (**g**) Violin plot of stomatal area. (**h**) Violin plot of stomatal number. Three asterisks (***) indicate statistically significant differences as determined by one-way ANOVA followed by Fisher’s LSD test (*p* < 0.001).

Statistical analysis of the stomatal length, width, area, and number on the adaxial and abaxial surfaces of wheat leaves revealed that the frequency distributions of these traits followed a normal distribution (Figure 3). The average stomatal length on the adaxial surface was 123.70 pixels, while the average stomatal length on the abaxial surface was 120.70 pixels (Figure 3a). Although the mean values are similar, the stomatal length on the adaxial surface is higher, suggesting that the stomata on the adaxial surface may be slightly longer in terms of structural development. The average stomatal width on the adaxial surface was 66.10 pixels, while the average stomatal width on the abaxial surface was 69.30 pixels (Figure 3b). These results indicate that the short-axis dimension of the stomata on the abaxial surface is larger, possibly due to its greater physiological role in gas exchange or transpiration regulation. The maximum stomatal area on the adaxial surface was 9083 pixels, while the maximum stomatal area on the abaxial surface was 8627 pixels (Figure 3c). Although the length on the abaxial surface is slightly smaller, the width and area are slightly larger. This may be due to differences in the stomatal opening angle or structural differences, which result in a larger area measurement. In a field of 1600 × 1200 pixels, the average number (22.80) of stomata on the adaxial surface was more than the average number (18.80) of stomata on the abaxial surface (Figure 3d).

Violin plots illustrating significant differences (*p* < 0.001) in stomatal traits between adaxial and abaxial surfaces across 210 wheat cultivars are shown in Figure 3. The stomatal length on the adaxial surface of leaves was significantly longer than that on the abaxial surface (Figure 3e); the stomatal width on the adaxial surface was significantly smaller than that on the abaxial surface (Figure 3f); the stomatal area on the adaxial surface was significantly smaller than that on the abaxial surface (Figure 3g); and the stomatal number on the adaxial surface was significantly greater than that on the abaxial surface (Figure 3h). Overall, the stomatal density on the adaxial surface is higher than that on the abaxial surface, while the stomatal size on the adaxial surface is smaller than that on the abaxial surface, indicating significant differences in the stomatal distribution strategy between the two leaf surfaces.

### 2.4. Stomatal Trait Correlation Analysis

**Figure 4:**
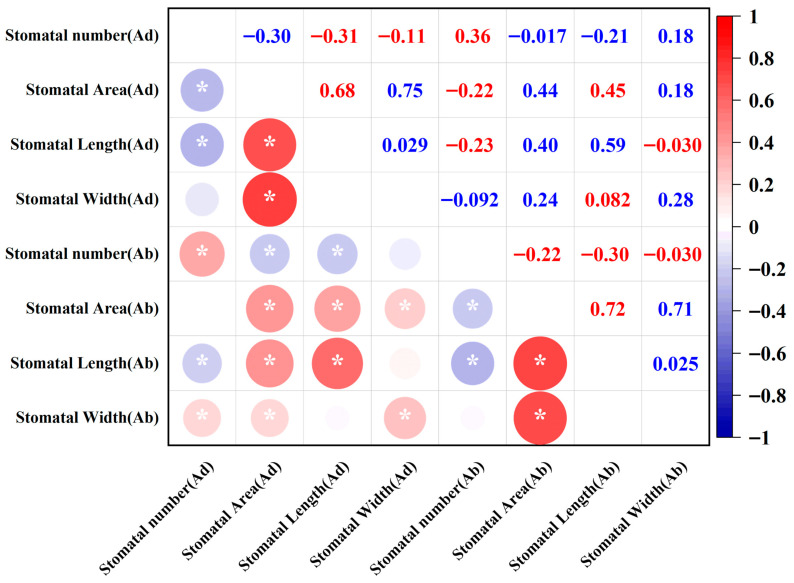
Correlation analysis of the 8 stomatal traits of the 210 wheat cultivars. (* means *p* < 0.05).

Correlation coefficient (r) displays the correlation relationships between eight stomatal traits at a significant level (*p* < 0.05), revealing the co-variation patterns of stomatal number, size, and area (Figure 4). The results indicate that the number of stomata on the adaxial surface is positively correlated with the number of stomata on the abaxial surface (r = 0.36) and the stomatal width on the abaxial surface (r = 0.18), suggesting a certain synchrony in the distribution of stomata on both sides of the leaf, with stomata on the abaxial surface tending to expand in high-density regions. Meanwhile, the number of stomata on the adaxial surface is negatively correlated with the stomatal length on both the adaxial surface (r = −0.31) and the abaxial surface (r = −0.21), reflecting a physiological trade-off relationship of “increased number—decreased length”. The number of stomata on the abaxial surface is also positively correlated with the number of stomata on the adaxial surface (r = 0.36) but negatively correlated with the stomatal length and area on both surfaces (r = −0.22~−0.30), suggesting that under high-density stomatal distribution on the abaxial surface, the structural development of individual stomata may be limited. Further analysis shows that the area of stomata on both surfaces is moderately positively correlated with the stomatal length and width on both surfaces (r = 0.68~0.75), indicating structural coordination between the stomata on both leaf surfaces. Additionally, the area of stomata on the abaxial surface is negatively correlated with the number of stomata on the abaxial surface (r = −0.22). Overall, the length and width of the stomata are closely related to the size of the stomata, while the morphological features of the stomata on the adaxial and abaxial surface have no direct correlation.

### 2.5. GWAS Results and Candidate Gene Identification

**Figure 5:**
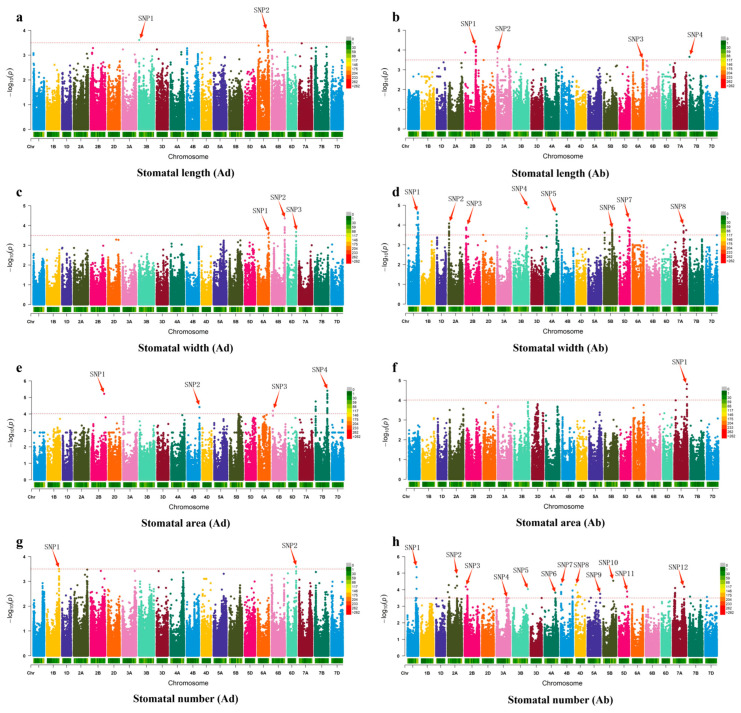
Genome-wide association study for wheat SNP markers of 8 stomatal traits of 210 wheat cultivars. (**a**–**h**) are Manhattan plots, in which the red arrows indicate significant associated loci.

The results of the GWAS revealed multiple genetic loci significantly associated with wheat leaf stomatal traits, covering key phenotypic indicators such as stomatal length, width, area, and number. For stomatal length on the adaxial surface, two significant loci were identified, located on chromosomes 3B and 6A (Figure 5a). For stomatal length on the abaxial surface, four loci were associated, located on chromosomes 2B, 3A, 6A, and 7B, suggesting that these chromosomal regions may contain key genes regulating stomatal longitudinal growth (Figure 5b). Three significant loci associated with stomatal width on the adaxial surface were located on chromosomes 6A, 6B, and 6D (Figure 5c), while the genetic regulation of stomatal width on the abaxial surface was more complex, with a total of eight significant loci detected, widely distributed on chromosomes 1A, 2A, 2B, 3B, 4A, 5B, 5D, and 7A, indicating multi-locus and multi-pathway regulation of this trait (Figure 5d). For stomatal area, the adaxial stomatal area was associated with four loci, located on chromosomes 2B, 4B, 6B, and 7B (Figure 5e). The abaxial stomatal area was associated with only one locus, located on chromosome 7A, suggesting that stomatal area development on the two sides of the leaf may be controlled by different genetic mechanisms (Figure 5f). Furthermore, the number of stomata on the adaxial surface was significantly associated with two loci on chromosomes 1B and 6D (Figure 5g), while the number of stomata on the abaxial surface was associated with as many as twelve significant loci, covering chromosomes 1A, 2A, 2B, 3A, 3B, 4A, 4B, 4D, 5A, 5B, 5D, and 7A (Figure 5h), indicating the complexity and diversity of genetic regulation for this trait.

This study further investigated 36 candidate genes significantly associated with stomatal traits identified through GWAS, using the Dr.Tom multi-omics data mining platform from BGI Genomics for functional annotation and biological significance analysis, with the aim of identifying potential functional genes and elucidating their roles in stomatal development and regulation. The annotation results indicate that two genes may directly participate in the physiological regulation of stomatal behavior. One gene, *TraesCS2B02G178000*, located on chromosome 2B, is predicted to be involved in stomatal movement and may affect gas exchange and water loss by regulating stomatal opening and closing signaling pathways, contributing to the plant’s response mechanism to environmental changes. Another significant gene, *TraesCS6A02G290600*, located on chromosome 6A, is involved in stomatal closure regulation and is hypothesized to regulate the physiological response of guard cells to environmental stresses such as drought or heat, thereby influencing the maintenance and regulation of stomatal dynamics (Appendix A). The identification of these two genes provides strong evidence for revealing the genetic basis of wheat stomatal traits and offers important candidate targets for further research into the molecular mechanisms of stomatal functional traits and breeding applications.

## 3. Discussion

### 3.1. Model Performance Testing and Comparative Experiments

The WSF proposed in this study has distinct advantages compared to previous models. In the current application of deep learning models for wheat stomata, two main types of models are commonly used: object detection models and semantic segmentation models. For object detection models, the research by [26] enhanced a CNN-based detection model by incorporating an attention mechanism, achieving a stomata recognition accuracy of 98.30%. However, the study used a general-purpose dataset created under ideal conditions with destructive sampling, which cannot effectively handle images with significant background interference. In contrast, the data in this study was collected from field conditions using non-destructive sampling, which includes interference from lighting, background impurities, and other real-world environmental factors, thus posing a greater challenge to the model’s generalization ability. This gives our model an advantage over those using general-purpose datasets. Similarly, some studies employed the YOLO architecture for stomata detection [27], but the detection framework returned bounding boxes, and the stomata located within these boxes still required conventional image processing methods for extraction. These conventional methods often miss detections and are ineffective in handling overlapping stomata or those that are too small. For semantic segmentation models, the U-Net network is known for its ability to process details and capture features at multiple scales, making it suitable for dynamic video streams of stomatal behavior [28]. However, U-Net’s decoder relies on skip connections, which preserve image details but may lead to poor segmentation precision for small objects like minute stomata. Its performance on static images is often unsatisfactory. On the other hand, SegFormer, using a Transformer architecture, is better at capturing long-range dependencies and global information, making it more suitable for segmentation tasks in complex backgrounds, particularly in handling fine stomata. To address the issue of inaccurate stomatal edge segmentation, we designed a CFPT. This module, by fusing features from multiple layers and refining them, effectively improves the completeness and accuracy of edge segmentation.

### 3.2. Genetic Regulation of Stomatal Traits and Application Prospects of GWAS

The stomata on both sides of the wheat leaf exhibit diverse and continuous variations in morphological traits such as length, width, area, and number, following normal distribution. This aligns with the genetic patterns of quantitative traits, indicating that stomatal traits are typical complex phenotypes controlled by multiple genes. As these traits play key roles in environmental adaptation, physiological metabolism, and yield regulation, their genetic basis warrants further exploration. Therefore, this high-quality phenotypic data provides a solid foundation for conducting GWAS. Combined with high-throughput genotypic data, these findings can be used to identify key functional genes that regulate stomatal development, density, and size, further assisting breeding decisions and providing theoretical and practical support for the selection of wheat varieties with high resistance and efficient water use.

Stomata serve as the passage for plants to exchange carbon and water with the environment, thus playing a crucial role in regulating their physiological activities. Therefore, the regulation of stomatal traits (such as morphology, number, size, and positioning on the leaf surface) can significantly affect the plant’s water use efficiency and, consequently, its yield [29]. Although the loci and signaling pathways controlling stomatal traits are generally highly conserved [30,31,32], significant functional differences in stomatal genes have been identified in various lineages [33]. These stomatal genes can influence traits related to water use, thereby affecting the plant’s resistance to abiotic stresses. Thus, screening and identifying stomatal traits that are responsive to drought phenotypes and molecular responses can help improve crop tolerance. Researchers have also screened the stomatal index of 539 wheat varieties [34] and used a wheat 660 K SNP chip for genotyping. Through GWAS analysis, they identified 130 SNPs significantly associated with the stomatal index of wheat seedlings, many of which overlapped with genes. Among the 130 gene loci detected in the study, 69 were positively specific and 61 were negatively specific, with almost no overlap between the SNPs associated with the front and back of the leaf stomatal indices. This finding is consistent with previous reports on wheat [35]. These results suggest that the genetic control of the stomatal index on the front and back of the wheat leaf is distinct.

In this study, we conducted a GWAS of stomatal-related traits (length, width, area, and number) in 210 wheat varieties and identified 36 SNP loci significantly associated with these traits. We found that compared with the adaxial surface of the leaves, there were more significantly associated gene loci on the abaxial surface, with 11 loci on the adaxial surface and 25 loci on the abaxial surface, respectively, and there was minimal overlap between them. Two genes—TraesCS2B02G178000 (on chromosome 2B) and *TraesCS6A02G290600* (on chromosome 6A)—were found to be associated with stomatal traits, involved in regulating stomatal movement and closure, respectively. By comparing these two candidate genes with their most clearly defined homologous genes, it was found that TraesCS2B02G178000 shares 82% identity with At1g22640 (SPCH) in Arabidopsis, a bHLH transcription factor that initiates stomatal lineage [36], and *TraesCS6A02G02G290600* is 75% identical to At5g53210 (EPF1), whose secreted peptide negatively regulates stomatal density and suppresses stomatal clustering [37]. This result is consistent with prior research. Furthermore, functional annotation of candidate genes revealed two potential loci involved in regulating stomatal movement and closure, validating the effectiveness of our WSF-powered GWAS.

### 3.3. The Generalization Ability of the WSF Model to Other Plants

**Figure 6:**
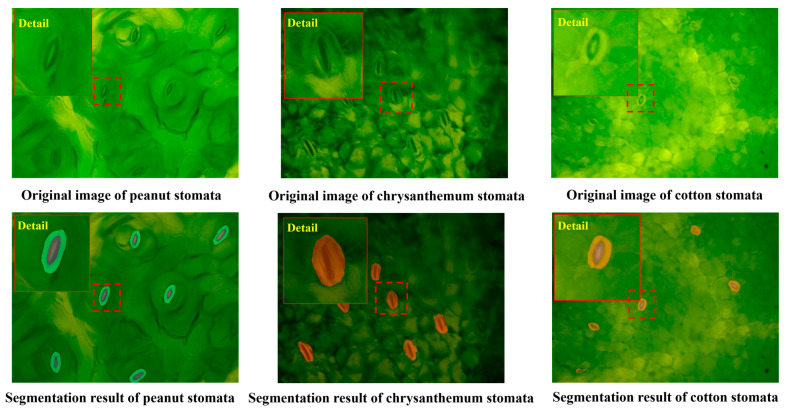
Segmentation results of the WSF model under different plants. (Red boxes in the figure are magnified stomata, making it easier to observe the segmentation effect of the model.).

In this study, the WSF model was also tested for generalization performance on three plant species, namely peanut, chrysanthemum, and cotton (100 images each) (Figure 6). Two evaluation metrics, precision and Mean Intersection over Union (mIoU), were employed, and the results are presented in Table 2. Among these species, Cotton demonstrated the best performance, with both precision and mIoU exceeding 83%, indicating that the model possesses strong transferability when applied to plants with similar stomatal morphology or relatively simple backgrounds. Peanut ranked second, achieving relatively high accuracy and good regional overlap, which suggests that the model also adapts well to leaves with relatively regular structures or moderate interference. In contrast, the performance on Chrysanthemum declined slightly due to its smaller stomata and more complex leaf surface texture. Chrysanthemum exhibited the lowest accuracy and segmentation quality, largely because its stomatal morphology differs markedly from the training data and background noise is strong, reflecting the challenges the model faces in cross-species applications with pronounced structural differences.

### 3.4. Limitations of the WSF Model and Future Research Directions

The WSF semantic segmentation model shows great promise in wheat stomatal analysis, enabling automated identification and segmentation of stomata in large-scale microscopic images and thus substantially improving research efficiency. Nevertheless, the model still presents several limitations in practical applications. First, its performance strongly depends on the quality and diversity of training data. Since most datasets are derived from high-quality laboratory-acquired images, segmentation accuracy often decreases under field conditions, where noise, uneven illumination, and leaf folding are common, revealing inadequate generalization ability. Second, the model may encounter recognition errors when applied to different wheat varieties, developmental stages, or under pathological influences, which limits its broader agricultural applicability. Furthermore, its ability to capture fine boundary details remains insufficient, leading to inaccuracies in quantifying stomatal aperture, thereby hindering deeper investigations into stomatal dynamics and physiological processes.

On the application side, although preliminary image-processing software integrating the WSF model has been developed to automate stomatal analysis, its functionalities remain relatively limited. Existing software typically supports only single-image processing and lacks efficient tools for large-scale batch or fully automated analysis, which is crucial for field monitoring and high-throughput experiments. In addition, the user interfaces are not yet sufficiently intuitive, posing challenges for agricultural researchers without computational backgrounds. More importantly, current software largely focuses on segmentation and area measurement, without establishing strong connections with physiological parameters such as stomatal density, aperture dynamics, transpiration rate, or photosynthetic efficiency. This limits its role in supporting crop stress resistance evaluation or breeding selection. Future efforts should therefore prioritize enhancing the model’s robustness and cross-environment adaptability, while promoting the development of intelligent, automated software platforms that integrate stomatal traits with physiological analysis, ultimately serving wheat stomatal research and agricultural production practices.

## 4. Materials and Methods

### 4.1. Stomatal Data Collection for 210 Wheat Varieties

A total of 210 wheat varieties (the names of all varieties are listed in Appendix A), including major wheat varieties and core germplasm resources from the Huang-Huai-Hai wheat region, were collected. The experimental materials were sown in late October 2023 at the Baima Teaching and Research Base of Nanjing Agricultural University (31.61° N, 119.18° E), Lishui District, Nanjing City, Jiangsu Province. The fertilizers used in the experiment included urea as the nitrogen fertilizer, monoammonium phosphate as the phosphorus fertilizer, and potassium chloride as the potassium fertilizer. The nitrogen fertilizer was applied at a rate of 240 kg hm^−2^, divided into basal fertilizer and jointing fertilizer with a 5:5 ratio. Both phosphorus and potassium fertilizers were applied at a rate of 120 kg hm^−2^ as a single application of basal fertilizer. A total of 210 experimental plots were used. Each plot was 1.50 m × 1.25 m in size, containing six rows of wheat with a row spacing of 0.25 m and 175 seeds in each row. The spacing between adjacent plots was 0.50 m horizontally and 0.45 m vertically. Field management was carried out according to standard high-yield cultivation practices.

Sampling for this study was conducted during the wheat regreening stage on 22 February 2024. To avoid edge effects in the experiment, three wheat plants with similar growth and morphology were selected from the second row of each plot from bottom to top. The top leaves of the main stems were collected, and the stomatal images of the wheat leaves were captured using an Anyty portable microscope (3R-WM601 USB series, BeiJing, China). The microscope includes an image acquisition button and a size adjustment knob. After connecting the Anyty microscope to a computer, turning the adjustment knob to change the focal length of the microscope can ensure the appropriate magnification for observing the wheat leaf stomata. In this study, stomatal images of wheat leaves were captured using the Anyty microscope, with a magnification of 550 times. The leaf was slowly moved so that the microscope was aligned with the center of the leaf, and the stomata were arranged as parallel as possible in the field of view. After photographing the stomata on the adaxial surface, the leaf was flipped to capture the stomata on the abaxial surface. The stomatal images were saved in JPG format. A total of 210 wheat varieties were photographed, with six images taken per variety (three for the adaxial surface and three for the abaxial surface), resulting in a total of 1260 stomatal images.

### 4.2. Construction of the Wheat Stomata Dataset

This study focuses on image segmentation for three target categories: wheat stomata (wheat_stoma), pores (pore), and background (background). During the data preparation phase, the ISAT Segment Anything tool was used to accurately annotate masks and optimize details for 1260 high-resolution images (1600 × 1200 pixels). To enhance data diversity, the research team implemented various data augmentation strategies, including random rotation, scaling transformations, and deformation processing, which ultimately expanded the sample size to 12,600 image sets (each set consisting of the original image and its corresponding mask). The fundamental principle of data augmentation lies in generating diverse samples through operations such as rotation, flipping, scaling, and noise injection, thereby enhancing the model’s adaptability to varying environments and disturbances. This process reduces overfitting and improves generalization.

For data splitting, the augmented dataset was scientifically allocated into training, testing, and validation sets in a 7:2:1 ratio. Specifically, 8820 image sets were designated for training, 2520 sets for testing, and 1260 sets for validation. This distribution scheme effectively ensured sample balance at each stage of model development. All image data were matched strictly using a file naming mechanism to ensure correspondence.

### 4.3. WSF Model Architecture

**Figure 7:**
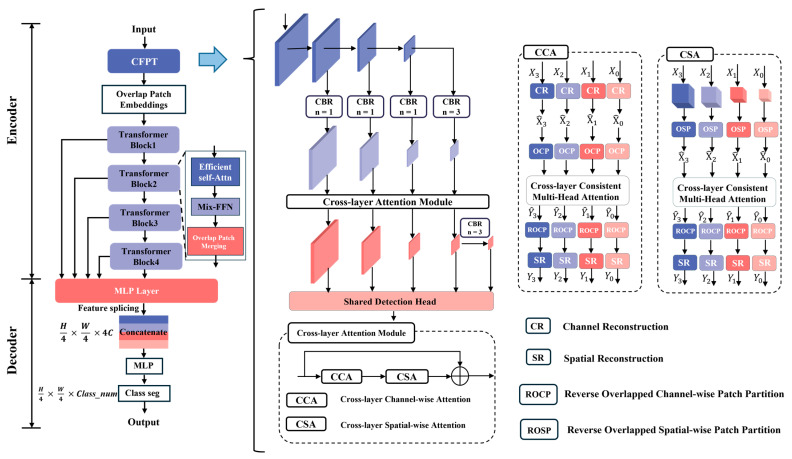
Schematic of the WSF model architecture. (Different colors represent modules with different functions, and the arrows between modules indicate a progressive relationship).

In this section, based on the SegFormer framework, we propose a semantic segmentation model for wheat stomata, named WSF (Figure 7). Accurate recognition of the stomatal edges is a key factor affecting the overall accuracy. However, there are several issues with the segmentation of wheat stomatal edges. First, the boundary between the stomata and the surrounding cell walls may have low contrast due to lighting conditions, imaging factors, or sample preparation methods (such as the leaf surface wax layer), making it difficult for traditional segmentation models to distinguish the edges accurately. Additionally, wheat stomata exhibit significant variation in shape and size (e.g., in open/closed states, different developmental stages), leading to inconsistent edge structures. Furthermore, small pores, intercellular spaces, or noise (e.g., dust or imaging artifacts) around the stomata may be misinterpreted as the stomatal edges, resulting in segmentation errors. To address these issues, we introduce a CFPT module to resolve these challenges. This module captures the subtle features of stomatal edges through multi-scale feature fusion, utilizes spatial/channel attention mechanisms to enhance the weight of edge regions, and combines the long-range dependency modeling ability of Transformers to optimize edge continuity.

The WSF model is composed of a hierarchical Transformer encoder and a lightweight decoder consisting of pure MLP. In the encoding phase, the raw image features are first processed by the CFPT module to enable cross-layer information interaction and edge-sensitive feature enhancement. Then, the model performs an overlapping block embedding operation on the input image, where the convolution kernel size is 7 × 7, the stride is set to 4, and the padding is 3, thus acquiring richer local information. Subsequently, the input is processed sequentially by four Transformer modules with progressively reduced resolutions, corresponding to down sampling ratios of 1/4, 1/8, 1/16, and 1/32, with channel dimensions of 64, 128, 320, and 512, respectively, to construct multi-scale semantic feature maps. The decoder then extracts features from these four stages. First, a 1 × 1 convolution is used to unify the channel number to 256 for subsequent fusion. After the feature maps are up sampled and aligned to a unified spatial size, they are concatenated along the channel dimension, then sent into multiple lightweight MLP branches for feature mapping and context enhancement. These branches operate in parallel to mine multi-scale semantic information. Finally, the outputs of all branches are concatenated and subjected to a convolution operation to complete channel compression and information fusion, resulting in a globally fused feature map. At the model output, a convolution layer matching the number of categories is set to generate the final semantic segmentation mask, achieving pixel-level precise recognition of wheat stomata, pores, and background regions.

The CFPT module divides the feature maps from different levels of the backbone network into four groups, each differing in spatial scale and channel number, reflecting the network’s perception of different semantic levels. To fully exploit the information complementarity among these features, CFPT module first uses a horizontal connection strategy to upsample high-level semantic features to the same spatial resolution as low-level features and align them in the channel dimension, enabling the fusion of features from different layers. The fused features are then fed into the cross-layer attention module, which consists of two key substructures: Cross-Layer Channel Attention (CCA) and Cross-Layer Spatial Attention (CSA). CCA focuses on the channel dimension and allows each channel token to perceive global context information across the entire spatial domain through a multi-scale adjacent feature interaction mechanism. Before this process, features at different scales undergo Channel Reconstruction (CR) to ensure compatibility in the channel attention computation by unifying the spatial resolution. CSA, on the other hand, operates on the spatial dimension, interacting features corresponding to the same spatial locations at different layers, enabling each spatial token to fuse semantic features from the channel dimension, thereby establishing more robust local context representations. Through these two attention paths, CFPT module achieves cross-layer feature fusion in both channel and spatial dimensions, constructing a new, semantically rich pyramid feature structure. Finally, this new multi-layer pyramid is input into a shared detection head, which predicts category information for all scale features, outputting a segmentation tensor of size (H, W, K), where H and W are the spatial resolution, and K is the number of target categories.

All experiments were performed on a desktop system featuring an Intel Core i9-14900 K processor(Intel Corporation, Santa Clara, CA, USA), 64 GB of system memory, and an NVIDIA GeForce RTX 4090 GPU with 64 GB VRAM. In the training process of the Wheat Stoma Former semantic segmentation model, carefully designed hyperparameter configurations were adopted to ensure a balance between convergence efficiency and model performance. In the training of the SegFormer model, *k*-fold cross-validation (e.g., *k* = 5) is employed to mitigate overfitting. The dataset is divided into five subsets, with each subset serving as the validation set in turn, while the remaining subsets are used for training. This iterative process ensures stable performance across varying data partitions, enhances generalization capability, and facilitates the selection of optimal hyperparameters. Specifically, the initial learning rate was set to 6 × 10^−5^, with a learning rate decay mechanism applied, reducing the learning rate by a decay factor of 0.8 after each training epoch to accommodate fine-tuning of parameters in the later stages of training. The model used a batch size of 8 and underwent 200 epochs of iterative training, with accuracy evaluated on the validation set after each epoch to monitor the model’s generalization performance and adjust strategies in a timely manner. The AdamW optimizer was used, which is an improved version of Adam that incorporates a weight decay regularization term to effectively mitigate overfitting and enhance training stability.

To comprehensively evaluate model performance, this study introduced two primary metrics: overall precision (Pre) and mean Intersection over Union (MIoU). Precision measures the model’s prediction accuracy for all categories, i.e., the proportion of true positives among all predicted positive samples; while MIoU calculates the ratio of intersection to union between predicted and ground truth regions and averages this value across all categories, reflecting the overall quality of spatial region segmentation. The computation of these two metrics is represented by Equations (1) and (2), which serve as key indicators for evaluating the precise segmentation capability of Wheat Stoma Former in stomatal regions.

(1) $$\mathrm{Pre}\;=\frac{{\mathrm{True}}_{p}}{{\mathrm{True}}_{p}+{\mathrm{False}}_{p}}$$

(2) $$\mathrm{MIoU}\;=\frac{1}{k+1}\;\sum_{i=0}^{k}\frac{{\mathrm{Area}}_{m}^{i}\cap {\mathrm{Area}}_{\mathrm{gt}}^{i}}{{\mathrm{Area}}_{m}^{i}\cup {\mathrm{Area}}_{\mathrm{gt}}^{i}}$$

Specifically, ${\mathrm{True}}_{p}$ and ${\mathrm{False}}_{p}$ represent the number of correctly and incorrectly classified pixels, respectively; ${\mathrm{Area}}_{m}\cap {\mathrm{Area}}_{\mathrm{gt}}$ and ${\mathrm{Area}}_{m}\cup {\mathrm{Area}}_{\mathrm{gt}}$ represent the number of intersection and union pixels of the model’s predicted mask and the manually annotated mask, respectively.

### 4.4. Wheat Stomatal Trait Extraction

In the wheat stomatal semantic segmentation task, the mask images generated by the semantic segmentation model enable precise localization and region separation of stomatal targets. Based on the mask images of the adaxial and abaxial surfaces of each wheat variety, the following four key phenotypic parameters can be extracted: stomatal length, stomatal width, stomatal area, and stomatal number. First, the mask images output by the semantic segmentation model are converted into binary images, where the stomatal region is white and the background is black. Then, the contours of each stomatal target are extracted using the Canny operator-based contour extraction method. Afterward, the minimum enclosing rectangle of each contour is fitted to obtain the stomatal length and width. Next, the number of pixels in each stomatal contour within the mask region is counted and multiplied by the physical area represented by a single pixel to obtain the stomatal area. Finally, the number of detected contours is used as the estimated value for stomatal count.

### 4.5. Genotyping and GWAS of Stomatal Traits in Wheat

In this study, a panel of 210 wheat accessions was genotyped using the Axiom^®^ Wheat660 high-density SNP array(Beijing, China), developed through a collaboration between the Institute of Crop Sciences, Chinese Academy of Agricultural Sciences, and Affymetrix Inc. (Santa Clara, CA, USA) [38]. This genotyping platform features over 95% effective marker coverage and ensures even genome-wide distribution. It allows the detection of 630,517 genetic variants, including both SNPs and small insertions/deletions (InDels). Initial genotype data underwent stringent quality control using Axiom Analysis Suite version 5.2 (Affymetrix, Santa Clara, CA, USA). Filtering criteria excluded SNPs with more than 10% missing data and minor allele frequencies (MAF) below 0.05. After quality control, a dataset comprising 409,976 high-confidence SNPs remained for genome-wide association analysis. SNPs were physically anchored to the wheat reference genome IWGSC RefSeq v1.0.

To explore the genetic basis of stomatal morphological traits on the adaxial and abaxial leaf surfaces, GWAS was conducted using the rMVP v0.99.26 software package [39]. The FarmCPU (Fixed and Random Model Circulating Probability Unification) model was employed to control false positives and increase detection power. Significant marker-trait associations were visualized using Manhattan plots. Candidate genes located near significant loci were identified using the WheatOmics multi-omics database [40]. Further functional annotation and biological relevance assessments of these genes were conducted via the Dr.Tom platform (https://biosys.bgi.com, accessed on 1 July 2023) provided by BGI Genomics.

### 4.6. Statistical Analysis

One-way ANOVA followed by Fisher’s LSD test was used to examine significant differences in stomatal traits difference between the adaxial and abaxial surfaces. The Loss curves, Precision curves, and MIoU curves of model training were all visualized using Python3.9. All the statistical analyses were conducted using SPSS 25, and other related figures were drawn using Origin 2024.

## 5. Conclusions

In this study, an automatic and high-throughput analysis pipeline for wheat stomatal phenotypic traits was established. The developed WSF semantic segmentation network was used to extract stomatal features, and the edge detection effect was enhanced through the CFPT module. The WSF model showed high accuracy, with a precision of 93.60% and an MIoU of 91.20%. The predicted values were highly correlated with the measured values, with the R^2^ value for stomatal number exceeding 0.92 and the R^2^ values for length and width both exceeding 0.84. The analysis of stomatal phenotypes in 210 wheat varieties revealed that the adaxial surface exhibited longer stomatal length but reduced width, resulting in smaller overall stomatal area compared to the abaxial side. Conversely, stomatal number showed an inverse pattern with higher frequency on the adaxial surface. Subsequently, GWAS was performed using 660 K SNP chip data, which identified 36 significant SNP loci on multiple chromosomes. Candidate genes such as *TraesCS2B02G178000* and *TraesCS6A02G290600* are related to stomatal regulation. These findings offer valuable insights into the genetic control of stomatal traits and provide targets for improving water use efficiency and drought tolerance in wheat breeding.

## Figures and Tables

**Table 1:** Performance comparison of the different methods.

Model Name	Precision	MIoU
WSF (our)	93.6%	91.2%
SegFormer	91.7%	90.1%
Deeplabv3+	89.5%	86.8%
UNet	87.7%	85.2%

**Table 2:** Performance comparison of the different plants.

Plant Name	Precision	MIoU
Peanut	81.1%	77.5%
Chrysanthemum	78.2%	72.3%
Cotton	83.6%	79.0%

## Data Availability

All data and materials are available upon request to QL (qingli@njau.edu.cn).

## Appendix A. Wheat Varieties Used in the Study

**Number**	**Variety Name**	**Number**	**Variety Name**	**Number**	**Variety Name**	**Number**	**Variety Name**	**Number**	**Variety Name**
1	Aikang 58	43	Jinan 16	85	Luomai 21	127	Shimai 22	169	Yannong 15
2	Baomai 10	44	Jinan 17	86	Luomai 26	128	Shiyou 20	170	Yannong 173
3	Beijing 10	45	Jining 16	87	Luomai 7	129	Shunmai 1718	171	Yannong 19
4	Boai 7023	46	Ji 5265	88	Machang 2	130	Sumai 188	172	Yannong 22
5	Chuanmai 22	47	Jimai 19	89	Mianmai 37	131	Sumai 3	173	Yannong 836
6	Chuanmai 36	48	Jimai 1	90	Mianmai 39	132	Tai 10604	174	Yannong 999
7	Chuanmai 42	49	Jimai 26	91	Xumai 32	133	Taikemai 31	175	Yangmai 10
8	Dekang 961	50	Jinan 2	92	Nannong 0686	134	Taikemai 33	176	Yangmai 13
9	Dexuan 1	51	Jinan 8	93	Ningmai 13	135	Taimai 1918	177	Yangmai 15
10	Emai 11	52	Jinan Ai 6	94	Ningmai 15	136	Tainong 19	178	Yangmai 158
11	Emai 12	53	Jinhe 9123	95	Ningmai 22	137	Taishan 1	179	Yangmai 16
12	Emai 15	54	Jinmai 31	96	Ningmai 24	138	Taishan 27	180	Yangmai 18
13	Emai 19	55	Jinmai 33	97	Ningmai 26	139	Taishan 28	181	Yangmai 1
14	Emai 6	56	Jinmai 47	98	Ningmai 9	140	Taishan 4	182	Yangmai 22
15	Fan 6	57	Jingdong 17	99	Ningnuomai 1	141	Taishan 5366	183	Yangmai 5
16	Fengde Cunmai 1	58	Jingdong 18	100	Nongda 211	142	Taitianmai 118	184	Yangmai 9
17	Fengkang 8	59	Jingdong 22	101	Qida 195	143	Tainong 24	185	Yumai 13
18	Gaoyou 503	60	Jinghua 9	102	Qimai 2	144	Wanmai 0066	186	Yumai 18
19	Guomai 301	61	Jingshuang 16	103	Qingnong 2	145	Wanmai 19	187	Yumai 2
20	Han 7086	62	Keyuan 088	104	Rumai 1	146	Wanmai 33	188	Yunhan 2233
21	Haoyou 2018	63	Laizhou 137	105	Ruihuamai 520	147	Wanmai 50	189	Yunhan 618
22	Henong 2063	64	Laizhou 953	106	Ruihuamai 523	148	Wanxi Mai 0638	190	Xumai 31
23	Henong 6049	65	Lianmai 2	107	Shannong 12	149	Wanfeng 269	191	Chang 4738
24	Henong 825	66	Lianmai 6	108	Shannong 15	150	Wannian 2	192	Zhemai 1
25	Heng 136	67	Lianmai 7	109	Shannong 20	151	Wennong 14	193	Zhenmai 12
26	Heng 4399	68	Liangxing 66	110	Shannong 205	152	Wennong 5	194	Zhenmai 168
27	Yangmai 20	69	Liangxing 77	111	Shannong 21	153	Wennong 17	195	Zhenmai 3
28	Hengguan 35	70	Lin Y8012	112	Shannong 22	154	Xinmai 16	196	Zhenmai 4
29	Hengza 102	71	Linmai 4	113	Shannong 24	155	Xinmai 18	197	Zhenmai 9
30	Huaimai 20	72	Lukenmai 9	114	Shannong 28	156	Xinmai 20	198	Zhengmai 004
31	Huaimai 22	73	Lumai 14	115	Shannong 29	157	Xinmai 9	199	Zhengmai 7698
32	Huaimai 28	74	Lumai 15	116	Shengxuan 3	158	Xinmai 296	200	Zhengmai 9023
33	Huaimai 29	75	Lumai 1	117	Shengxuan 6	159	Xingmai 18	201	Zhongmai 175
34	Huaimai 30	76	Lumai 21	118	Shiluan 02-1	160	Xingmai 7	202	Zhongmai 9
35	Huaimai 33	77	Lumai 22	119	Shimai 26	161	Xumai 30	203	Zhoumai 12
36	Huaimai 6	78	Lumai 5	120	Shimai 28	162	Xumai 33	204	Zhoumai 18
37	Jimai 19	79	Luyuan 502	121	Shi 4185	163	Xuzhou 24	205	Zhoumai 23
38	Jimai 20	80	Lunxuan 987	122	Shijiazhuang 407	164	Xuzhou 438	206	Zhoumai 24
39	Jimai 21	81	Luohan 11	123	Shijiazhuang 8	165	Xuzhou 8	207	Zhoumai 26
40	Jimai 22	82	Luohan 13	124	Shimai 12	166	Yanfu 188	208	Zhoumai 27
41	Jimai 229	83	Luohan 2	125	Shimai 15	167	Yannong 0428	209	Zhoumai 32
42	Jimai 23	84	Luohan 9	126	Shimai 18	168	Yannong 1212	210	Zinong 033

## Appendix B. Significant Associations Markers Based on GWAS of Stomatal Traits

**Table:**

Trait	Marker	Loc	Var	−log10	SnpEffect_GeneID	Gene Annotation
Length (Ad)	SNP1	Chr3B:24820702	C->T	3.50	TraesCS3B02G048900	oxidoreductase activity
Length (Ad)	SNP2	Chr6A:522154021	G->A	3.86	TraesCS6A02G290600	cellular response to calcium ion, regulation of stomatal closure
Length (Ab)	SNP1	Chr2B:579569265	C->A	4.01	TraesCS2B02G407900	integral component of membrane transmembrane transport
Length (Ab)	SNP2	Chr3A:15558194	G->A	3.90	TraesCS3A02G030300	integral component of membrane
Length (Ab)	SNP3	Chr6A:571935441	A->C	3.50	TraesCS6A02G338600	transaminase activity cellular amino acid metabolic process
Length (Ab)	SNP4	Chr7B:52781	G->A	3.65	TraesCS7B02G000200	ATP binding, ATPase activity, coupled to transmembrane movement of substances
Width (Ad)	SNP1	Chr6A:609452855	C->A	3.63	TraesCS6A02G400000	iron–sulfur cluster binding, metal ion binding
Width (Ad)	SNP2	Chr6B:710007244	C->T	4.36	TraesCS6B02G451100	aspartic endopeptidase activity, intramembrane cleaving, membrane protein proteolysis
Width (Ad)	SNP3	Chr6D:468806891	A->G	3.67	TraesCS6D02G396300	RNA polymerase
Width (Ab)	SNP1	Chr1A:531677694	T->C	4.58	TraesCS1A02G342900	ATPase activity, coupled to transmembrane movement of substances, ATP binding
Width (Ab)	SNP2	Chr2A:36021015	G->T	4.08	TraesCS2A02G073500	UDP-glycosyltransferase activity
Width (Ab)	SNP3	Chr2B:54532482	A->G	3.75	TraesCS2B02G094100	protein kinase activity, ubiquitin–protein transferase activity, ATP binding, oxidoreductase activity
Width (Ab)	SNP4	Chr3B:705591855	C->T	3.84	TraesCS3B02G462700	integral component of membrane
Width (Ab)	SNP5	Chr4A:624015701	T->G	4.54	TraesCS4A02G344100	O-methyltransferase activity; protein dimerization activity; P-nucleus; hormone-mediated signaling pathway; methylation; Q-positive regulation of transcription, DNA-templated;
Width (Ab)	SNP6	Chr5B:457122696	T->C	3.64	TraesCS5B02G271400	RNA binding; intracellular membrane-bounded organelle; RNA modification
Width (Ab)	SNP7	Chr5D:549858298	C->G	4.23	TraesCS5D02G536800	plant-pathogen interaction; environmental adaptation; organismal Systems
Width (Ab)	SNP8	Chr7A:548367285	T->C	3.68	TraesCS7A02G375500	calcium ion binding; RNA binding; methyltransferase activity; RNA methylation
Area (Ad)	SNP1	Chr2B:703976259	G-> T	5.22	TraesCS2B02G507100	ATP binding, ADP binding, defense response
Area (Ad)	SNP2	Chr4B:656816645	G-> T	4.41	TraesCS4B02G370800	cation transmembrane transporter activity
Area (Ad)	SNP3	Chr6B:32330944	C->G	4.19	TraesCS6B02G068800	raffinose alpha-galactosidase activity, carbohydrate metabolic process
Area (Ad)	SNP4	Chr7B:641806970	G->T	5.41	TraesCS7B02G377100	extracellular region
Area (Ab)	SNP1	Chr7A:675287935	G->T	4.57	TraesCS7A02G684200	integral component of membrane
Number (Ad)	SNP1	Chr1B:667223816	G->A	3.50	TraesCS1B02G447600	RNA binding, metal ion binding; nucleus
Number (Ad)	SNP2	Chr6D:471016984	G->A	3.59	TraesCS6D02G402800	amRNA binding, DNA binding, metal ion binding; poly(A)+mRNA export from nucleus
Number (Ab)	SNP1	Chr1A:503377978	C->A	4.75	TraesCS1A02G311900	DNA-binding transcription factor activity, RNA polymerase II-specific
Number (Ab)	SNP2	Chr2A:52763048	T->C	4.28	TraesCS2A02G102500	other types of O-glycan biosynthesis, Glycan biosynthesis and metabolism; oxidoreductase activity
Number (Ab)	SNP3	Chr2B:153128770	A->G	3.57	TraesCS2B02G178000	regulation of stomatal movement
Number (Ab)	SNP4	Chr3A:535189608	G->T	3.50	TraesCS3A02G301400	rRNA 5′-end processing, rRNA processing
Number (Ab)	SNP5	Chr3B:822681495	C->T	4.03	TraesCS3B02G602700	MAPK signaling pathway-plant
Number (Ab)	SNP6	Chr4A:590590535	C->T	3.82	TraesCS4A02G283700	amino sugar and nucleotide sugar metabolism
Number (Ab)	SNP7	Chr4B:21556722	G->A	4.30	TraesCS4B02G029400	amino sugar and nucleotide sugar metabolism
Number (Ab)	SNP8	Chr4D:11914487	C->T	4.29	TraesCS4D02G026900	amino sugar and nucleotide sugar metabolism
Number (Ab)	SNP9	Chr5A:682898348	T->C	3.75	TraesCS5A02G521600	magnesium ion binding, terpene synthase activity; terpenoid biosynthetic process
Number (Ab)	SNP10	Chr5B:550156642	C->T	4.54	TraesCS5B02G563800	Aminoacyl-tRNA biosynthesis; DNA binding, sigma factor activity
Number (Ab)	SNP11	Chr5D:450860102	G->T	3.89	TraesCS5D02G380500	ribosome biogenesis in eukaryotes; GTPase activity, GTP binding
Number (Ab)	SNP12	Chr7A:116506563	T->C	3.66	TraesCS7A02G160400	MAPK signaling pathway-plant, Plant hormone signal transduction, Plant-pathogen interaction

Note: Candidate genes associated with stomatal regulation are marked in red.
